# Prevalence and molecular identification of *Nematodirus helvetianus* in camels in Iraq

**DOI:** 10.14202/vetworld.2021.1299-1302

**Published:** 2021-05-25

**Authors:** Amer Rasool Alhaboubi, Ali Issa Fadhil, Shehala Rasool Feidhel

**Affiliations:** Department of Parasitology, College of Veterinary Medicine, University of Baghdad, Baghdad, Iraq

**Keywords:** camel, internal transcribed spacer, *Nematodirus* spp

## Abstract

**Background and Aim:**

Camels from the central part of Iraq are infected with multiple parasitic diseases that have an economic impact by decreasing meat and milk production. This study aimed to evaluate *Nematodirus* spp. in camels (*Camelus dromedarius*).

**Materials and Methods:**

The study animals consisted of camels slaughtered in the central area of Iraq at the Al-Najaf slaughterhouse. All ages and sexes of camels were examined. Worms were recovered and identified microscopically. For molecular characterization, two Iraqi *Nematodirus* spp. partial ribosomal genes (ITS1 and ITS2) were sequenced and submitted to the NCBI database.

**Results:**

Of 160 camels tested, 29 were infected with *Nematodirus* spp. (18.13%). Twenty-one nematodes containing the *Nematodirus* genes were identified in the small intestines of naturally infected camels. BLAST analysis revealed 88.1% sequence similarity with that of *Nematodirus helvetianus* isolated in China and 87.2% similarity with *N. helvetianus* isolated in the United States.

**Conclusion:**

The prevalence of *N. helvetianus* warrants the use of anti-helminthic drugs for these animals and a rationale for future control strategies to prevent the transmission of this infection to other livestock.

## Introduction

Camels are generally used for transportation, but are also considered a source of meat, milk, leather, and wool since ancient times [1]. Their biological and anatomical features as well as their demeanor make them well adapted for dry climates [2].

Parasites represent a significant challenge to animal health. They primarily affect normal physiology and productivity because they decrease food consumption and cause clinical symptoms such as diarrhea. However, most helminthiases are asymptomatic or without clinical manifestation, thus the herd may appear normal, despite performing below its full potential [3,4]. The genus *Nematodirus* belongs to trichostrongylids which are the most common helminths and are considered the most important nematodes that have an economic impact by afflicting ruminates, including camels and camelids [5,6]. *Nematodirus* is a nematode of the small intestine of large and small ruminants and exhibits a direct life cycle. The genus *Nematodirus* consists of more than 45 species distributed in domestic ruminants worldwide [6,7]. *Nematodirus helvetianus* is frequently a parasite of cattle (generally named the twisted wireworm). The adults are 10–25 mm in length and coiled in a spiral-like fashion. The eggs are characterized by a large thick shell and contain 4-8 cells (blastomeres). They are shed into the feces and do not hatch until the larva 3 has developed inside. These parasites are found in dry or frigid climates where other genera die because of desiccation [8].

In Iraq, a few studies have established camelid gastrointestinal nematodes, in particular, *Nematodirus* spp. Altaif [9] identified the infection with helminths in the gastrointestinal (GI) tract. This study aimed to evaluate *Nematodirus* spp. in camels (*Camelus dromedarius*).

## Materials and Methods

### Ethical approval

Samples collection and transformation were permitted by the Iraqi Ministry of Agriculture Regulation, and the study research protocols were approved by the College of Veterinary Medicine, University of Baghdad laboratory standards.

### Study period and location

The study started from early September till the end of December 2018. Samples were procured from the slaughterhouse and processed at the Parasitology Department in the College of Veterinary Medicine at Baghdad University.

### Samples collection

Small intestine samples were obtained directly from animal carcasses after slaughtering in Al-Najaf abattoir (in the central province of Iraq). A total of 160 small intestine samples of camel were included (130 males and 30 females) in the study.

### Morphological examination

Samples were sheared longitudinally and examined directly for any nematodes attached to the mucosal surface or with digested contents. Initially, the isolated worms were washed with distilled water to prevent damage and directly examined under a light microscope to maintain its integrity, and lactophenol was used to observe the structures. Captured images, scanning, and morphometric data were obtained using an Olympus L × 70 microscope (Olympus America, Inc., Center Valley, PA) using Spot Software 5.2 Macro photography © 2016 For further examinations, the nematodes were preserved in 70% ethanol. The morphological identification of the adult male nematodes was made based on Soulsby [8].

### DNA extraction

A teen adult female *Nematodirus* spp. (morphologically identified) was randomly selected for DNA extraction [WizPrep™ gDNA Mini Kit (Cell/Tissue), Wizbio Co., Korea)]. Electrophoresis on 2% agarose gel was done to confirm the integrity and purity of the extracted DNA. The DNA concentration was determined by spectrophotometry (NanoDropND-1000 Spectrophotometer, Wilmington, DE, USA). The DNA concentrations were stored at −20°C.

### Primer selection

Ribosomal DNA is a common target for the design of probes or selected markers. For the identification of different types of bursate nematodes, primers were synthesized to target the ITS1-5.8s-ITS2 region of the ribosomal RNA gene of *Nematodirus* *oiratianus* based on the GenBank sequence. The forward primer was 5′-GTAGGTGAACTGCGGAAGGATCATT-3′ and the reverse primer was 5′-TTAGTTTT CCTCCGCT-3′ [10].

### Polymerase chain reaction (PCR)

The amplification reactions (PCR mix and amplification profile) were performed according to the manufacturer’s instructions (GoTaq® G2 Hot Start Green Master Mix Co., USA) Table-1. The thermocycler was run under the condition as per Table-2.

**Table-1:** Polymerase chain reaction master mix used for DNA amplification.

Component	Volume	Final concentration
GoTaq at Hot Start Green Master Mix. 2X	12.5 µL	1X
10 µM Primer F gene	2.5 µL	1.0 µM
10 µM Primer R gene	2.5 µL	1.0 µM
DNA ( ∼100 ng)	2 µL	200 ng
Nuclease-free water	Up to 25 µL	N.A
Total		25 µL

GoTaq® G2 Hot Start Green Master Mix Co., USA

**Table-2:** Thermocycler conditions set for the polymerase chain reaction.

Step	Time	Temp	No.
Initial denaturation	3 min	95°C	1
Denaturation	15 s	95°C	35 cycles
Annealing	30 s	54°C	
Polymerization	1 s	72°	
Final extension	5 min	72°C	1
Infinity	----	4°C	----

### Sequencing

Amplicons of approximately 870 bp from five worm samples were submitted for Sanger sequencing (Sanger Sequencer, NICEM, South Korea). The resulting sequences were analyzed by MEGA6.0 (https://www.megasoftware.net/) [11] and adjusted to a corresponding fragment length. Similarities to the ITS1-ITS2 gene sequences in the GenBank database were determined using the NCBI BLAST program [12]. The sequences were deposited into the GenBank database and accession numbers (MT850129 and MT705244) were assigned. The sequences were aligned using Clustal W2 software (https://www.ebi.ac.uk/Tools/msa/clustalw2). The final sequence alignment with the isolates found in GenBank was used to construct a maximum likelihood tree in DIVEN (https://indra.mullins.microbiol.washington.edu/DIVEIN/diver.html) using the default setting.

## Results and Discussion

### Prevalence finding

**Figure-1:**
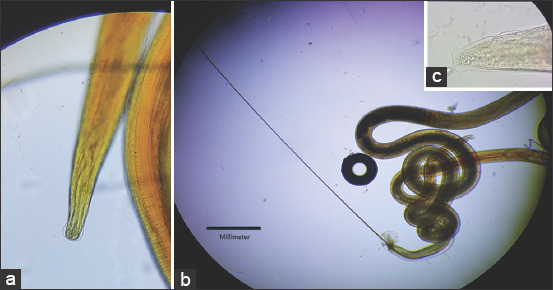
*Nematodirus helvetians* (a) Male and female anterior end. (b) Male posterior end. (c) Female posterior end.

This study revealed the presence of the adult male and female nematodes in the small intestine of the camel, Figure-1 [13]. *Nematodirus* spp. is usually listed as a cattle nematode, though it has been found in many other domestic and wild ruminants, including sheep and camels [3,14-17]. There are few studies on the adult helminths in camels and the previous studies have focused on the eggs or the whole larva [18-20]. The data showed a total *Nematodirus* spp. infection rate of 18.13% (Table-3). A previous morphometric analysis recorded a measurement of 14 mm for the adult male body and a 21.5 mm length for the female adult parasite [14]. Male camels showed a higher infection rate (24/130; 18.46%) compared with females (5/30; 16.67%) (Table-3).

**Table-3:** The infection rate of the *Nematodirus* spp. in small intestine of camel.

Animals	Number	Infected	%
Male	130	24	18.46
Female	30	5	16.67
Total	160	29	18.13

A prevalence study indicated that a total of 210 camels were slaughtered in Shiraz (South of Iran) that were infected with the Nematodirine subfamily species. *N. helvetianus* (15.24%) was associated with the highest infection rate followed by *Nematodirus spathiger* (14.29%) and *N. cameli* (5.7%), and of which were localized in the small intestine [17]. In a subsequent study of 144 adult camels slaughtered in abattoirs in Iran, the infection rate of the species varied as follows: *N. helvetians* (0.7%), *N. abnormalis* (2.1%), *N. dromedarii* (3.5%)*, N. oiratianus* (4.9%), and *N. spathiger* (6.9%) [3].

## Molecular findings

**Figure-2:**
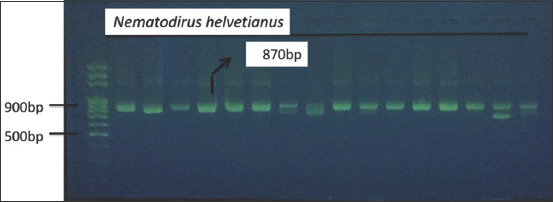
Ethidium bromide-stained agarose (2%), presenting: Polymerase chain reaction products of ~870 bp showing the actual size of the large subunit ribosomal RNA gene *Nematodirus helvetianus*.

DNA amplification of the large subunit of the ribosomal RNA gene yielded a single fragment of approximately 870 bp in 15 samples (Figure-2). Unfortunately, of the submitted PCR amplicons, sequencing data for only two samples were obtained, however, they consisted of short nucleotide fragments. Both sequences (MT705244.1; 448 bp and MT850129.1; 207 bp) exhibited high similarity to *N. helvetianus* gene sequences.

### Sequencing analyses

A part of the large subunit of the ribosomal RNA gene of *Nematodirus* spp. was sequenced. The sequences obtained in this study were compared with those in the GenBank database using BLAST analysis. The results revealed 88.1% similarity with *N. helvetianus* isolated in China (KC580751.1) and 87.2% similarity with *N. helvetianus* isolate in the USA (AF194141.1).

**Figure-3:**
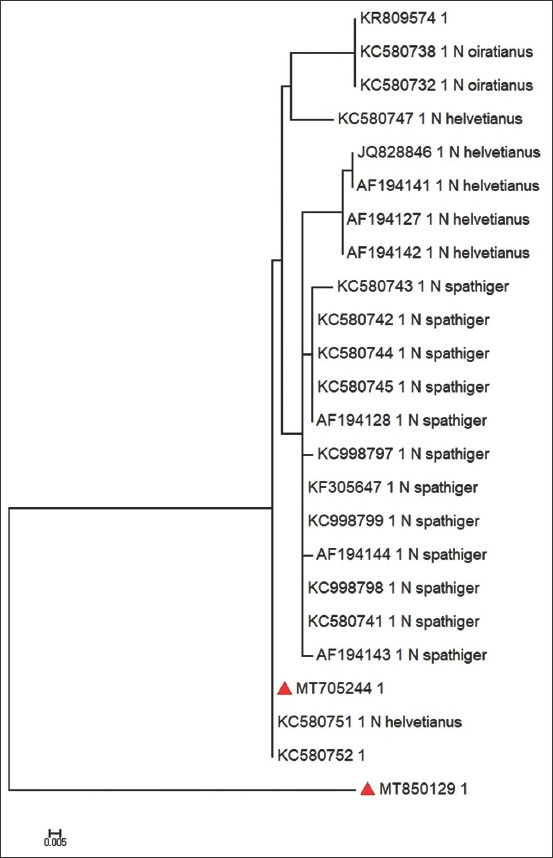
Maximum likelihood phylogenetic tree based on the ITS1-ITS2 rRNA gene sequences. *Nematodirus helvetianus* found in the camels in this study are indicated by red triangle.

The two sequences were aligned with 22 corresponding ITS-ITS2 gene sequence and were closely related taxa. The final aligned sequence was 206 bp after trimming the sequence as required for continual fragmentation. A maximum-likelihood phylogenetic tree was generated from the final alignment of the sequences and showed three main clades of *Nematodirus* infecting a variety of host ruminants (cattle, sheep, and camels). The first clade contained the Iraqi sequences and two isolates of *N. helvetianus* identified in China. The second consisted of three clusters, but branched into *N. helvetianus* (from the USA), *N. spathiger*, and *N. oiratianus*. The second Iraqi isolates grouped with the three main clades. The comparison revealed variations among and within geographic regions. The phylogenetic tree topology shows similarities and differences among the globally registered sequences of *N. helvetianus* and our registered isolates (MT850129 and MT705244) (Figure-3).

A general analysis of the genetic similarity using rRNA sequences data showed the greatest pairwise similarity between *N. spathiger* and *N. helvetianus*, *Nematodirus battus*, and the nucleotide sequence of ITS-2 rRNA (3.9-24.7%) [21]. The previous studies used the ITS1/ITS2 genes (ribosomal) as well as the cytochrome b gene (mitochondrial) as reliable molecular markers for different nematode and protozoan parasites in Iraqi camels and cattle [22]. This is the first morphological (male) and molecular (female) study to identify *N. helvetianus* in the Iraqi camel [23,24]. However, the lack of data within the geographic locations renders the comparison with the corresponding references insufficient to address genetic variation. Therefore, future studies with similar or different genetic markers such as mitochondrial DNA are needed.

## Conclusion

Camels can be infected with gastrointestinal tract helminths including *N. helvetianus* which known to infect cattle, sheep, and other ruminants. General protection programs and control strategies are necessary to enhance animal health and productivity as camels may transmit diseases to other livestock and humans.

## Authors’ Contributions

ARA, AIF and SRF: Participated in the study design. ARA and AIF: Worked on the morphological examination and molecular finding with analysis. SRF: Drafted the manuscript. ARA: Revised the manuscript. All authors read and approved the final manuscript.
